# Placebo Intervention Enhances Reward Learning in Healthy Individuals

**DOI:** 10.1038/srep41028

**Published:** 2017-01-23

**Authors:** Zsolt Turi, Matthias Mittner, Walter Paulus, Andrea Antal

**Affiliations:** 1 Department Clinical Neurophysiology, University Medical Center, Georg-August University, Göttingen, Germany; 2 Department of Psychology, University of Tromsø, Tromsø, Norway

## Abstract

According to the placebo-reward hypothesis, placebo is a reward-anticipation process that increases midbrain dopamine (DA) levels. Reward-based learning processes, such as reinforcement learning, involves a large part of the DA-ergic network that is also activated by the placebo intervention. Given the neurochemical overlap between placebo and reward learning, we investigated whether verbal instructions in conjunction with a placebo intervention are capable of enhancing reward learning in healthy individuals by using a monetary reward-based reinforcement-learning task. Placebo intervention was performed with non-invasive brain stimulation techniques. In a randomized, triple-blind, cross-over study we investigated this cognitive placebo effect in healthy individuals by manipulating the participants’ perceived uncertainty about the intervention’s efficacy. Volunteers in the purportedly low- and high-uncertainty conditions earned more money, responded more quickly and had a higher learning rate from monetary rewards relative to baseline. Participants in the purportedly high-uncertainty conditions showed enhanced reward learning, and a model-free computational analysis revealed a higher learning rate from monetary rewards compared to the purportedly low-uncertainty and baseline conditions. Our results indicate that the placebo response is able to enhance reward learning in healthy individuals, opening up exciting avenues for future research in placebo effects on other cognitive functions.

Observational and interventional approaches are concomitantly used to study brain networks and their putative contributions to certain brain functions^1^. While the former approach is necessary to characterize the spatiotemporal patterns of neural activity, the interventional approach constitutes an important step towards facilitating causal inference^2^. Non-invasive brain stimulation (NIBS) interventions offer the possibility to induce transient perturbations in intact human brain networks by using electromagnetic induction or electrical current, while minimizing possible health risks, financial costs and ethical concerns^3^,^4^,^5^.

Transcranial direct current stimulation (tDCS) is the most frequently employed research tool in studies that use electrical current as a NIBS technique^3^. It passes constant, low intensity current between two or more electrodes attached to the intact scalp. Depending on the polarity, the externally applied constant current can increase or decrease the spontaneous firing rate (i.e., cortical excitability) of the stimulated brain regions by depolarizing or hyperpolarizing resting membrane potentials^3^. Although most of the commercially available and certified tDCS devices are equipped with a double-blind operation mode, a large number of studies still use a single-blind study design, no blinding at all or inadequate blinding^6^. In these cases, the impact of intentional and unconscious preferences, bias and expectations of the participants as well as of the researchers can possibly lead to an overestimation of its effectiveness. While several tDCS methods have been shown to be effective in the motor and cognitive domains, the variability in the response rate to tDCS is relatively high and it is unclear how much of the observed effectiveness is solely due to a placebo effect^7^,^8^,^9^.

The placebo effect is a complex psychobiological response to the application of a simulated intervention^10^,^11^,^12^,^13^. A prominent conceptual framework focusing particularly on the dopamine (DA)-mediated placebo mechanisms suggests that the placebo response can be considered a special case of a reward anticipation process, characterized by a neural overlap between anticipating the putative beneficial effects of the treatment and anticipating rewards^14^,^15^. In line with this hypothesis, substantial placebo-induced release of DA was detected both in the nigrostriatal and mesolimbic DA-ergic pathways, key regions in reward processing^16^,^17^,^18^,^19^, using positron emission tomography (PET)^14^,^20^,^21^,^22^,^23^,^24^.

Although the overwhelming majority of placebo studies focused primarily on placebo analgesia, a recent study of the placebo effects on reward learning indicates that there may be a common neurobiological mechanism of placebo effects across multiple domains^24^. Using pharmacological, neuroimaging and computational modeling measures, this study revealed that placebo medication enhances reward learning in Parkinson’s disease (PD) patients, and that the effects were driven by the DA-ergic system. However, due to the characteristics and the pharmacological treatment of PD patients, it remains elusive whether this cognitive placebo effect was mediated by placebo-induced expectations or also partly due to conditioning by the pharmacological treatment that the PD patients were receiving. Since PD patients are characterized by pathological DA-ergic network functioning^25^, they may constitute a subpopulation that is particularly responsive to the placebo intervention.

In the current study we therefore examined whether placebo interventions associated with different verbal manipulations would be capable of enhancing reward learning in healthy individuals. This active placebo intervention was applied using sham protocols of NIBS for the following reasons. First, in the past decade the application of tDCS has become increasingly popular in neuroscience research investigating both healthy individuals and patient populations. Recent years have seen an enormous increase in the use of tDCS extending well beyond academic and clinical applications to, e.g., do-it-yourself brain stimulation communities and professional athletes^26^,^27^. However, the placebo-inducing potential of tDCS is under-researched, although the placebo effect induced by medical devices are as strong or even superior to orally administered placebos^28^. Second, despite its extensive application in the motor and cognitive domains, its precise mechanism of action remains elusive, and the high inter-individual variability in the response to tDCS has yet to be further investigated^7^,^8^,^9^. Third, the ‘fade-in, short-stimulation, fade-out’ protocol is a well-characterized sham condition for tDCS, that produces a mild degree of cutaneous discomfort, such as itching, tingling or burning sensations on the scalp but does not induce physiological after-effects. Hence, for the purpose of the present study tDCS was an ideal candidate for an active placebo intervention (i.e., inducing mild sensations).

In addition to tDCS, we also employed another NIBS device called transcranial near infrared laser stimulation (tNILS) as a passive placebo (i.e. inducing no sensations). Earlier findings have shown that tNILS can alter cellular respiration processes by increasing mitochondrial adenosine triphosphate synthesis and cellular nitric oxide release^29^. Due to its effects at the molecular and cellular levels, tNILS has been shown to induce vasodilation that is beneficial in wound healing and in reducing inflammatory processes^29^. Similar to tDCS, tNILS can modulate cortical excitability in the human motor cortex^30^. Although tNILS has been applied in neurorehabilitation^31^, our knowledge about its effect on cognitive functions is limited^32^. The rationale for the application of tNILS was first to provide different treatment characteristics for the repeated-measures design both for the participants and the operator, and second, to avoid the possibility that participants or the operator would encounter earlier scientific publications on the effect of tDCS on cognitive functions that would contradict the verbal manipulation.

Earlier studies reported that placebo effects are most pronounced when the volunteers believed that they are certain to receive an active intervention^33^,^34^,^35^,^36^. Therefore, in the present experiment, participants were subjected to two active placebo conditions, in which the participants were misleadingly informed that they would receive an active intervention. Crucially, however, the two placebo conditions differed with regard to the declared certainty about the effectiveness of the intervention. In the low-uncertainty condition, the intervention (tDCS and tNILS) was introduced as a well-established, performance-enhancing method and was claimed to certainly improve the participants’ cognitive performance. In contrast, in the high-uncertainty condition, the active placebo stimulation was introduced as an experimental method whose effectiveness was not yet proven. In reality, all participants received 30 s of sham tDCS as an active placebo using a randomized, triple-blind (i.e., participant, operator and analyst were unaware of the conditions^37^), cross-over study design.

We considered two competing hypotheses regarding the possible outcomes of our study. First, it is conceivable that participants will show a stronger placebo effect when instructed that the effect is more certain, i.e., better learning will be observed in the low-uncertainty than in the high-uncertainty condition. This expectancy hypothesis is based on the assumption that the verbal manipulation will differentially increase the participants’ expectations, which eventually results in stronger cognitive placebo effects in the low-uncertainty condition, similar to the observations in the domain of placebo analgesia^33^,^35^,^38^. However, the opposite hypothesis, i.e., that the placebo effect would be stronger in the condition where treatment efficacy is less certain, is also conceivable in the light of the placebo-reward theory^14^,^15^. Based on the findings in PD patients, this theory proposes that the development of the placebo effect is tightly coupled to the activation characteristics of the reward circuit to reward uncertainty^15^. It has been shown that the tonic activation of midbrain DA neurons and, correspondingly, reward uncertainty are related by an inverted U-shape function with strongest activation when uncertainty is maximized^15^,^22^. If cognitive placebo effects are mediated by the activation of the reward circuit, then the low-uncertainty condition (according to the expectancy hypothesis) or the high-uncertainty condition (according to the placebo-reward hypothesis) could conceivably increase endogenous midbrain DA release, which could modulate reward processing and eventually lead to a more pronounced placebo effect.

Therefore, we hypothesized that if the placebo intervention were indeed capable of modulating reward learning in healthy individuals similar to PD patients^24^, we would be able to detect its behavioral consequences in a monetary reward-based reinforcement-learning task. This task is a well-characterized paradigm, known to strongly depend on midbrain DA levels^17^,^39^,^40^,^41^,^42^,^43^. In order to gain further insight into the underlying mechanisms of how placebo affects reinforcement learning, we applied a computational, temporal-difference reinforcement learning technique using a hierarchical Bayesian estimation procedure. The learning rate parameter used in our model represents the degree to which participants were able to learn from the reward prediction error, the difference between the obtained and the predicted reward. If the active placebo intervention(s) did indeed activate the reward circuitry, we would expect an increased learning rate from monetary rewards (i.e., optimized learning from gains), similar to the behavioral consequences of pharmacological studies in which the DA-ergic network was triggered by medication^44^,^45^.

## Results

**Figure 1:**
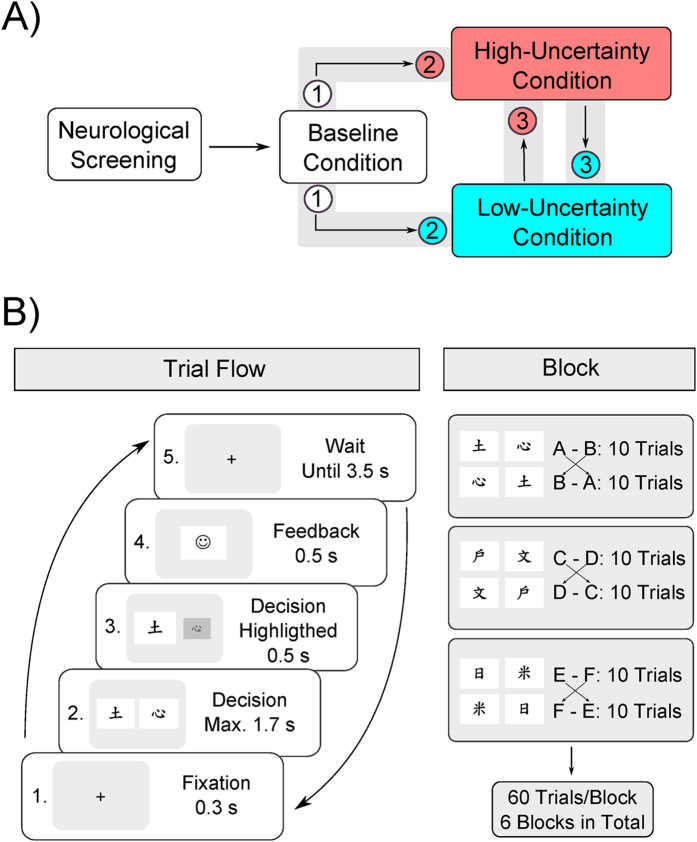
Flow of the volunteers through the conditions (**A**) and trials (**B**). (**A**) After a neurological screening, all participants first entered a baseline condition followed by the two randomly ordered placebo conditions (low- and high-uncertainty). (**B**) Participants performed a standard reinforcement-learning task^39^ where they had to select the better of two Chinese symbols.

We monitored participants’ performance during three repetitions of a standard reinforcement-learning task using unrelated stimulus sets^39^. In this task, the participants had to choose between pairs of Chinese symbols that were probabilistically mapped to a reward^42^. To optimize their monetary outcome, participants had to learn the stimulus that yielded a reward with maximum probability. Participants took part in three sessions. In each experimental session, participants had to learn three distinct stimulus pairs that had different reward contingencies (80/20%, 70/30% and 60/40%, see Fig. 1B). The first session served as a baseline condition and the other two sessions were placebo conditions, in which the participants’ belief about the effectiveness of the intervention was manipulated by verbal instruction (low-uncertainty vs. high-uncertainty condition, see Fig. 1A and Methods for details). Placebo intervention was delivered by an active sham protocol of tDCS (see Methods for details) that is insufficient to induce physiological changes in the brain, but capable of inducing substantial cutaneous sensations^46^,^47^.

For data analysis, we exclusively used Bayesian methods because of their many advantages compared to traditional null-hypothesis testing in general^48^,^49^ and in particular because they allow a hierarchical integration of the computational reinforcement-learning model^50^. We report our results in terms of posterior mean and the 95% highest-density intervals (HDIs), which give the range in which the estimated parameter is expected to be located with probability 0.95 given the model assumptions.

### Placebo increases objective performance measures

We analyzed learning performance by submitting the accuracy and reaction time data from the learning part of the experiment to independent Bayesian hierarchical generalized regression models. We included trial number, condition (baseline; low- uncertainty, LU; high-uncertainty, HU) and pair number (1, 2, 3) as predictors and let the intercept vary by subject (Methods for details). Non-informative priors were placed on all parameters (see Table 1 for a summary of the models’ results).

The results from this analysis clearly show substantially increased performance compared to baseline, both with regard to accuracy (*b*_LU_ = 0.217, HDI = [0.146, 0.279] and *b*_HU_ = 0.435, HDI = [0.370, 0.503]) as well as to reaction times (*b*_LU_ = −0.116, HDI = [−0.124, −0.107] and *b*_HU_ = −0.145, HDI = [−0.153, −0.136]). Even though new and randomized stimuli were used in each of the three sessions, it is possible that these effects are at least partly driven by a between-session practice effect from baseline to the (randomized) placebo conditions. Crucially, however, the performance boost was greater (evidenced by a greater increase in accuracy and a greater reduction in RT) in the high-uncertainty condition than in the low-uncertainty condition as indicated by the non-overlapping HDIs for all parameters.

**Figure 2:**
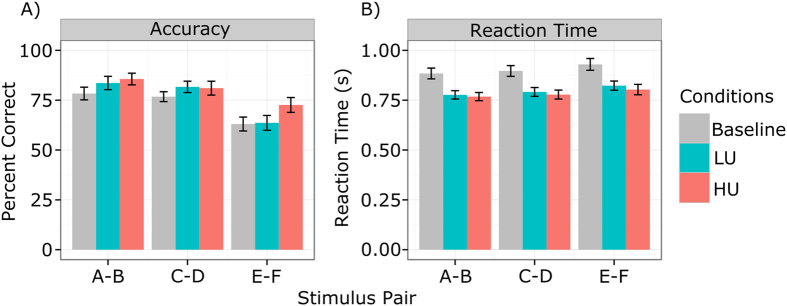
Descriptive statistics for accuracy (% correct, (**A**)) and reaction time (in s, (**B**)) for each condition and stimulus pair. Error bars show the standard error of the mean (SEM). Accuracy increases and reaction time decreases in both placebo conditions, but more strongly in the high-uncertainty condition. Accuracy and RT also depend on stimulus pair, reflecting their increasing degree of difficulty due to lower stimulus-reward contingencies. HU: high-uncertainty; LU: low-uncertainty placebo conditions

We also found the expected effect of trial number on accuracy, *b* = 0.144, HDI = [0.127, 0.160], and RT, *b* = −0.043, HDI = [−0.045, −0.041], indicating learning over the course of the experiment. Finally, the effect of pair number, reflecting increasing difficulty due to lower reward contingencies, was in the expected directions for accuracy, *b* = −0.497, HDI = [−0.532, −0.463] and reaction times, *b* = 0.023, HDI = [0.019, 0.027]. Descriptive statistics of the performance data are summarized in Fig. 2.

### Uncertainty modulates learning rate parameters of the reinforcement-learning model

To investigate the possible impact of our placebo manipulations on the learning rates, we fitted a computational reinforcement- learning model specifically designed for this task^39^ (see Methods). This model was designed to capture two independent learning-rate parameters, *α*_*G*_ and *α*_*L*_, reflecting the participants’ propensity to learn from gains and losses, respectively^39^,^51^. It has been suggested^39^ that learning from losses and gains might be implemented in different underlying neural mechanisms. In a data-driven model-comparison, we found that this more complex model clearly outperforms a model that does not separate between gains and losses, ΔWAIC = 539.93. We fitted a hierarchical Bayesian version of this model (see Methods) and estimated the effects of the placebo intervention on the learning rate parameters *α*_*G*_ and *α*_*L*_ and the noise parameter *β*.

**Figure 3:**
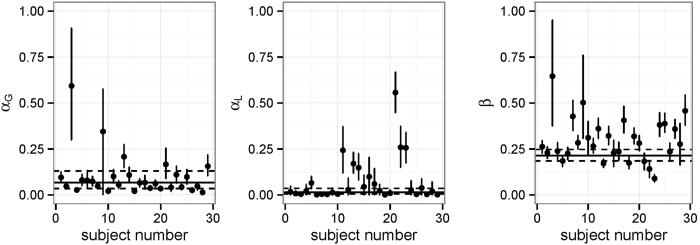
Individual parameter estimates for the baseline condition for the reinforcement-learning model. Dots indicate the posterior mean and the flankers the 95% highest-density interval (HDI). Black solid line indicates the mean of the group-level distribution and dashed lines the 95% HDI for that parameter.

**Figure 4:**
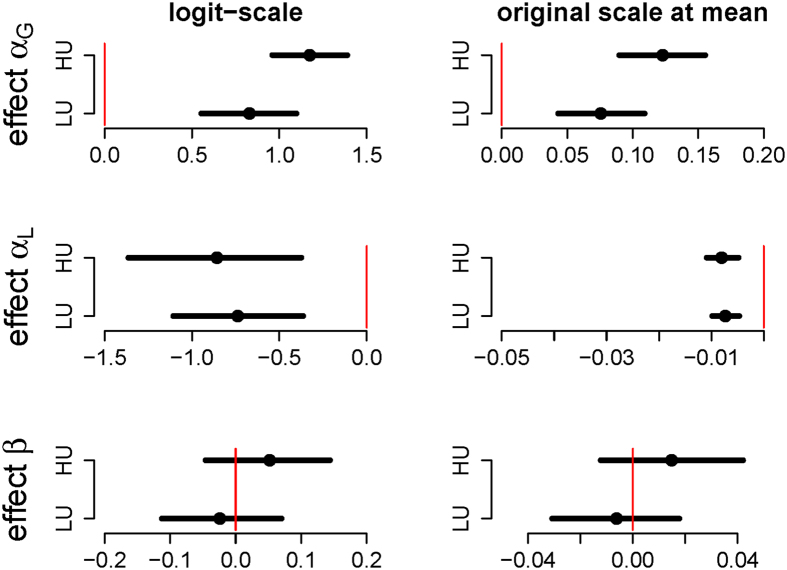
Estimates of the effects of the placebo interventions on the model parameters *α*_*G*_, *α*_*L*_ and *β* (rows), on the logit (left), and original scales at the mean (right). Circles represent the posterior mean, solid bars show the corresponding HDI. The thin red line indicates zero effect.

At the group level, the estimates for the learning rates and the noise parameter were in a reasonable range with the learning- rate for losses being lower than that for gains, 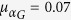, HDI = [0.03, 0.12], 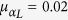, HDI = [0.004, 0.03] and *μ*_*β*_ = 0.21, HDI = [0.18, 0.25] (see Fig. 3). The effect of the placebo interventions was restricted to the learning-rate parameters (see Table 2: Learning from gains was improved in both placebo conditions and substantially more so in the high-uncertainty condition (*δ*_LU_ = 0.83, HDI = [0.55, 1.10] and *δ*_HU_ = 1.17, HDI = [0.96, 1.39], *P(δ*_HU_ > *δ*_LU_) = 0.99), mirroring the results from the analysis of accuracy and reaction times. In addition, both placebo manipulations induced a substantial, but small, decrement of the learning rate from losses, *δ*_LU_ = −0.74, HDI = [−1.11, −0.36], *δ*_HU_ = −0.86, HDI = [−1.37, −0.37] but the two conditions did not differ from one another. Finally, there was no effect on the noise-parameter *β (δ*_LU_ = −0.02, HDI = [−0.11, 0.07]; *δ*_HU_ = 0.05, HDI = [−0.04, 0.14]). To better interpret the size of the effects, which are give on the logit/log scales, we transformed them to the original scale when the intercept was at the mean (Fig. 4). While the effect on *α*_*G*_ was quite strong (≈0.1), it was much weaker for the *α*_*L*_ parameter (≈−0.01).

### Subjective expectation and experience is unaffected by placebo intervention

**Figure 5:**
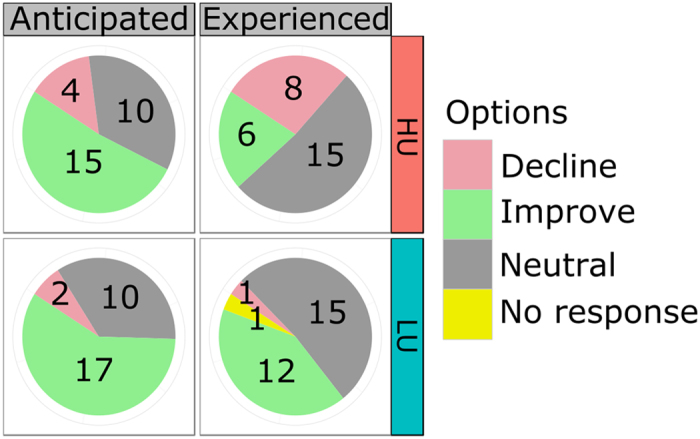
The anticipated and experienced impact (decline, no change or an improvement) of the intervention reported by the participants in the high-uncertainty (HU) and low-uncertainty (LU) placebo conditions.

We also asked our participants whether they anticipated or experienced a positive, negative or no impact of the intervention on their performance. The results of the responses to these questions are summarized in Fig. 5. We submitted these data to a softmax-regression model^49^ using type of question (anticipated vs. experienced changes) and condition (low- and high-uncertainy) as well as their interaction as predictors (see section 6 for details). None of the predictors showed a substantial deviation from zero (see Table 3). There was no strong evidence of a main effect of question (anticipated vs. experienced changes in performance), *β*_2,decline_ = −1.53, HDI = [−4.72, 1.50], *β*_2,improve_ = −0.92, HDI = [−2.10, 0.21] (both HDIs include zero). There was no apparent effect of condition (low- vs. high-uncertainty), *β*_1,decline_ = 0.50, HDI = [−1.66, 2.77] and *β*_1,improve_ = −0.06, HDI = [−1.25, 1.15] and neither an interaction of question and condition showed *β*_3,decline_ = 2.02, HDI = [−1.41, 5.64], *β*_3,improve_ = −0.51, HDI = [−2.25, 1.24]. All of the HDIs include zero and we cannot conclude with confidence that subjective anticipations or experiences were influenced by the placebo intervention.

**Figure 6:**
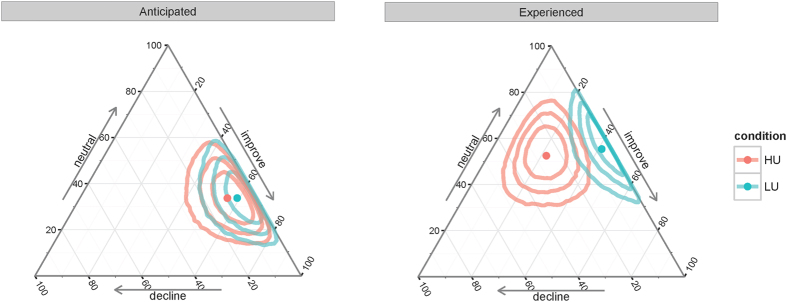
Ternary plots of the participants’ probability (in %) to respond that they anticipated or experienced a decline, no change, or an improvement due to the placebo intervention. Dots show the posterior mean, outlines show the 50%, 80% and 95% Highest-density regions (HDRs).

To make the results of this model more interpretable, we calculated the associated probabilities p for each of the conditions and questions. These values indicate estimated probabilities for each condition to anticipate or experience a decline, no change, or an improvement in performance due to the placebo intervention. In both conditions, anticipated changes in performance were similar and mainly neutral or positive, *p*_LU_ = (0.07, 0.34, 0.59) and *p*_HU_ = (0.10, 0.34, 0.56), (Fig. 6, left). Regarding the experienced performance changes, the estimated probabilities show a somewhat stronger tendency to experience no change, and more “decline”, and fewer “improve” ratings in the HU condition *p*_LU_ = (0.03, 0.56, 0.41), *p*_HU_ = (0.26, 0.52, 0.22), (see Fig. 6, right). However, the 95% highest-density regions (HDRs) for these estimates overlap (corresponding to the inconclusive HDIs on the interaction parameter) and any putative difference must therefore be interpreted carefully.

### Self-reported arousal was unaffected by placebo intervention

To exclude the possibility that our results reflect the effect of a general arousal due to the presence of an intervention rather than a genuine placebo effect, we asked our participants to rate their arousal before and after each experimental session on a scale from one to ten. This data was subjected to a similar analysis as the one above.

In general, our participants reported being quite awake as indicated by the fact that most responses were located at the upper end of the scale, *μ*_0_ = 7.22, HDI = [6.56, 7.86]. Participants’ responses after an experimental session were generally lower than before, *β*_after_ = −0.91, HDI = [−1.71, −0.11] (approximately one point on the ten point scale). There was no apparent effect of either condition (*β*_LU_ = −0.32, HDI = [−1.05, 0.51], *β*_HU_ = −0.18, HDI = [−0.91, 0.62]) or a modulation of the before/after effect due to condition (*β*_LU×after_ = 0.10, HDI = [−1.05, 1.19], *β*_HU×after_ = −0.11, HDI = [−1.23, 1.02]. We conclude that there is no apparent effect of the placebo stimulation on subjectively experienced arousal.

## Discussion

We investigated whether verbally manipulating the participants to be more or less certain about the effectiveness of the applied placebo NIBS would influence the performance of healthy individuals in a reinforcement-learning task, i.e., induce a cognitive placebo effect. We found that performance was increased in both low- and high-uncertainty conditions but more strongly in the high-uncertainty condition. Our Bayesian analysis revealed that participants in the high-uncertainty condition learned better from monetary rewards, compared to the low-uncertainty and training conditions. We found no difference in the subjective expectations reported in the two sessions.

The question of whether the placebo response is confined to placebo analgesia or patient-reported symptoms of various diseases has provoked a considerable debate in the placebo literature during the past years^52^. To date, only a handful of studies have investigated the placebo effect in the cognitive domain^24^,^53^,^54^, and even fewer studies deal with healthy persons^55^,^56^,^57^,^58^. Only one study investigated the possible biological systems involved in the generation of cognitive placebo effects by examining PD patients^24^. The study showed that placebo medication enhanced reward learning. The results of neuroimaging and computational measurements led to the conclusion that the observed cognitive improvement was mediated by the DA-ergic system^24^. Nevertheless, it remained unclear whether verbal manipulation alone can induce a cognitive placebo effect, or whether this is mediated by a combination of verbal manipulation and conditioning. PD patients have typically undergone several months or even years of DA-ergic treatment, and prior experience with the beneficial effect of the pharmacological intervention is thought to promote positive preconceived expectations from the treatment, also known as conditioning. Thus, PD patients with a malfunctioning DA-ergic system might constitute a special patient population that is particularly responsive to placebo intervention in reward learning, since the same DA-ergic system is targeted by the DA-restoring pharmaceuticals. We therefore only recruited healthy individuals who had no prior experience with DA-ergic medication.

Our findings constitute the first behavioral evidence of a cognitive placebo effect in a reinforcement-learning task in healthy individuals induced by the combined application of verbal manipulation and sham protocols of NIBS interventions. Furthermore, the pattern of our behavioral and computational modeling results are conceptually compatible with the findings in the reward learning in PD patients reviewed above, as both studies observed behavioral improvement in reward learning and optimized learning rate parameters for rewards. We extended earlier findings on cognitive placebo effects to include healthy adults and to NIBS interventions. This is of particular importance given that our results can potentially be applied to a wide range of laboratory situations.

In order to induce physiological or behavioral after-effects, tDCS needs to be applied at least for three minutes at 1 mA, because shorter stimulation protocols only induce immediate effects^59^. Beyond the primary physiological effects that constitute the main focus of most of the NIBS studies, the application of tDCS even at relatively low intensities (starting from about 0.4 mA) induces intensity- and electrode size-dependent cutaneous sensations, also known as the secondary induced effect of tDCS, which requires the use of an active sham control^47^,^60^,^61^. The most frequently applied sham protocol for tDCS is the so called ‘fade-in, short stimulation, fade-out’ protocol^46^, which is thought to lack any physiological or behavioral after-effects due to the low stimulation intensity and short stimulation duration^59^. Still, the present experiment has demonstrated that even an active sham protocol of tDCS is able to modify cognitive performance in healthy individuals when it is combined with verbal manipulation. Although tDCS is the most popular electrical NIBS intervention, its placebo-inducing potential in the cognitive domain was unknown until now. This is an important knowledge gap given that its precise mechanism of action is not fully understood, and that there is a relatively high response variability^7^,^8^,^9^. We therefore posit that, in addition to other variance-inflating factors such as individual brain anatomy and brain state before and during stimulation, individual differences in the susceptibility to the placebo intervention are important factors to consider when elucidating for which individuals tDCS would be effective.

Our results highlight the importance of careful study designs and data acquisition in NIBS studies. We argue that uncertainties regarding the expected efficacy of tDCS can be conveyed to the participants via the consent forms or during the interactions between participants and researchers in a manner similar to our verbal manipulations, and can induce cognitive placebo effects and increase effectiveness of the treatment. As most of the ISO certified stimulators are capable of operating in double-blind study mode, performing randomized double-blind placebo-controlled tDCS studies does not require additional investments, and double-blind designs should be required. Further, tDCS studies may consider assessing preconceptions, expectancy and former knowledge on the part of the participants about the expected efficacy of tDCS as our results indicate that these preconceptions might shape the resulting effect of the intervention. In spite of best efforts, blinding efficacy cannot always be maintained throughout the stimulation^6^. We therefore further recommend collecting information on the blinding efficacy and the perceived treatment if blinding is inadequate.

Our behavioral and computational data showed greater learning enhancement in the high-uncertainty condition compared to the low-uncertainty condition. It may be that the perceived improvement relative to baseline, or the less notable difference relative to the low-uncertainty condition led the participants to believe that the stimulation was, in fact, effective. In the domain of placebo analgesia, Rief and colleagues^35^ compared the effect of passive placebo (i.e., causing no sensations) and active placebo (i.e., causing minor sensations, such as in the present study) when the probability of receiving a drug was 0, 0.5 and 1. The authors found that active placebo, which is conceptually equivalent to our low- and high-uncertainty conditions substantially increased the placebo effect compared to the passive placebo in the 0.5 probability condition. It is assumed that the participants attributed the minor side-effects associated with the active placebo treatment to having received an active treatment. Thus, in the context of the present study the improvement in the high-uncertainty condition relative to the training session together with the cutaneous sensations may have implicitly biased the participants toward believing that the behavioral improvement was due to the applied intervention. The experience of improvement in the high-uncertainty condition may further reinforce the participants’ belief while performing the cognitive task that the intervention was effective and actually enhanced their performance. This hypothesized effect may be analogous to observations in the pain domain, where the relief from pain is rewarding in itself^62^.

Our main finding of enhanced learning in the high- relative to the low-uncertainty condition is based solely on behavioral data and computational modeling results, and we did not measure brain activity directly. Nevertheless, we can speculate that a biologically plausible explanation for our results is that the placebo stimulation together with the verbal instructions, triggered DA release in the midbrain as observed earlier in PD patients^24^. Because the placebo responses access the DA-ergic network which neurophysiologically overlaps with the neural network involved in reinforcement-learning, the increased DA levels would hypothetically be able to induce enhanced task performance and optimize learning rate from monetary rewards. This effect has been observed in earlier studies in which DA-ergic signaling was enhanced pharmacologically^44^,^45^. Since the tonic response of the midbrain DA-ergic neurons is maximized when reward uncertainty is high, we posit that the higher uncertainty triggered endogenous midbrain DA release that could have in turn improved the learning performance. As our study lacks physiological or neuroimaging data to support our assumptions, future research concentrating on the biological mechanisms of cognitive placebo effects in healthy participants will provide more insight into the role of DA-level dynamics in this phenomenon.

Reducing performance in the reinforcement learning task to the model-free learning system might be an oversimplification, since there are different neurobiological systems that support performance in the this task. We showed that placebo influenced the model-free learning system, but our results do not exclude the possibility that the cognitive placebo effect might be modulated by more explicit decision strategies (model-based system) that are not captured by the model used here, or by attentional influences that might be modulated by uncertainty^63^. In addition, DA is only one of the neurotransmitters involved in generating the placebo response, as opioid, endocannabinoid and serotonergic systems have also been shown to be contributing factors although in different domains of interest^13^,^64^,^65^,^66^,^67^. Moreover, although we found no difference in the subjectively reported arousal level between any of the applied conditions, differences in the stress level induced by the different stimulation setups could have influenced the observed placebo effect^68^. Future studies are needed to address the impact of arousal and stress on the cognitive placebo effect.

The fact that we observed no difference between the two placebo conditions in terms of subjectively reported expectations is somewhat surprising, given the explicit differences in the instructions. Together with the clear behavioral differences between the conditions, this intriguing pattern of results needs to be investigated in future studies using more in-depth measures of subjective expectations. Our experiment assessed explicit expectations with a single question: “What do you think? Will the stimulation change your performance?” posed immediately after the instruction and before the stimulation. This simple question might have been too coarse to detect differences in expectancy. Furthermore, our approach did not differentiate between different dimensions of expectancy such as strength and confidence. For instance, it would be interesting to study whether expectancy confidence or strength of expectancy was modulated by the placebo intervention. In addition, expectancy shifts and confidence levels could be assessed multiple times during the experiment to evaluate the development of these values in the course of performing the tasks. It is also possible that the introduction of the NIBS device in the high-uncertainty condition distracted the participants in such a fashion that they were less able to concentrate on the verbal manipulation. In order to investigate this scenario, additional experiments are required that employ the NIBS device without the verbal manipulation. Alternatively, future studies could combine verbal manipulation with conditioning by surreptitiously manipulating the feedback given the participants in each trial, which would make the task subjectively easier or more difficult. We expect that such a procedure would have the potential to influence the participants’ expectations to a larger extent.

Another limitation is the gender of the participants, as the present study recruited only males to avoid gonadal steroid mediated changes in DA signaling observed in an earlier study with females^69^. By controlling estradiol and progesterone levels between the different conditions, future studies that include females could investigate the placebo response in both genders^70^,^71^. Furthermore, the present findings are confined to the placebo effect, and it has yet to be investigated whether a cognitive nocebo effect, a decrease in learning performance following the administration of an simulated intervention, could be similarly evoked by verbal instruction^53^,^68^,^72^. Finally, careful replication studies are needed, preferably performed by independent laboratories, in order to confirm our findings and investigate whether the present results can be generalized to various other cognitive tasks, personality traits, genetic factors or different cultures^11^,^53^,^57^,^73^.

Overall, our behavioral and computational modeling results support the idea that placebo is a powerful phenomenon to improve reward learning even in healthy individuals. We showed that a cognitive placebo effect in healthy individuals does not necessarily require a conditioning procedure but can be evoked purely by verbal manipulation combined with sham NIBS protocols. Our results open up novel and exciting avenues of future research on the effects of cognitive placebo and highlight the importance of carefully designing informed consent forms, controlling contextual information and social interaction in NIBS studies.

## Methods

### Participants

Thirty participants were recruited for the experiment via online advertisement. All participants signed an informed consent form. The experiment was approved by the Ethic Committee of the University Medical Center Göttingen and was performed in accordance with relevant guidelines and regulations. Twenty-nine volunteers completed the study (mean age: 23.3 ± 2.95 yrs; mean years of education: 15.8 ± 2.35 yrs), as one participant did not return after the first session. In order to avoid menstrual cycle-dependent alterations in reward-related neural processing^69^, only male participants were recruited. None of the participants was familiar with Chinese or Japanese characters, similar to the procedure of an earlier study^39^. Before being finally recruited, the volunteers went through a neurological screening performed by a neurologist at the Department Clinical Neurophysiology, University Medical Center Göttingen, who was blinded to the genuine purpose of the study. Exclusion criteria included a history of current medical, neurological or psychiatric illnesses including epilepsy, drug and/or alcohol addiction, and the presence of metal implants in the head, neck and chest. Participants were asked about prior experience with brain stimulation. Only one participant had had prior experience with transcranial magnetic stimulation (TMS) and one other with transcranial direct current stimulation (tDCS). In both cases, the stimulation had been delivered to the motor cortex.

### Placebo induction and blinding procedure

The operator responsible for participant recruitment and for data collection was blinded to the purpose of the study and to the stimulation parameters during the time course of the entire study. The efficacy of blinding was confirmed verbally at the end of the study by the primary investigator. The operator was a native German medical student (22 year-old, female), naïve to tDCS and transcranial near infrared light stimulation (tNILS) studies and had no prior experience with non-invasive brain stimulation research. Before the start of the study, the operator received several weeks of special training in data collection. As the emotional characteristic of the experiment was not manipulated, the operator was instructed to use a neutral style during the consultation. In addition, the researcher performing the formal data analysis was unaware of which of the three conditions was the baseline, low- and high-uncertainty condition, respectively, until the data analysis was completed. This approach, often referred to as a triple-blind design^37^, was taken to minimize the cognitive biases that have recently been shown to be pervasive in scientific data acquisition and analysis^74^.

### Treatment characteristics

Following the protocols of previous procedures, we used expectation-inducing verbal instructions with the purpose of manipulating the uncertainty about the declared effectiveness of the stimulation^22^,^38^. We implemented a low-uncertainty (LU) and a high-uncertainty (HU) condition. In the LU condition, participants were given the information that they would receive a combined stimulation of both tDCS and tNILS that had previously been shown to be an effective intervention to improve human cognition. In the HU condition, participants were informed that they would receive a tDCS intervention whose effect was not yet experimentally proven.

We used different treatment characteristics in the HU and LU conditions. Sham tDCS was used in the HU while both sham tDCS and an inactive tNILS device were used in the LU. For tNILS, four inactive laser needles were selected, a fact unknown both to the operator and to the participants. The laser needles were carefully focused on the left DLPFC, and were fixed using a metal crown during the stimulation period and removed afterwards. In addition, the operator was required to wear goggles as protection from the “laser beam”, while the participants were asked to close their eyes during the stimulation. Participants received only sham tDCS in both treatment conditions (HU, LU). The anodal electrode was placed over the F3 location corresponding to the left dorsolateral prefrontal cortex (DLPFC), the cathode over the F4 location corresponding to the right DLPFC. The stimulation current was 1 mA following the fade in (15 s), short stimulation (30 s) fade out (15 s) approach^46^. With this procedure, the stimulation-induced cutaneous sensations were physically equivalent in the LU and HU conditions. Therefore, any difference in performance change detected between the two conditions cannot be explained by different stimulation setups.

The rationale for the different treatment characteristics was first to provide different conditions for the repeated-measures design both for the participants and the operator, and second, to avoid the possibility that participants or the operator might encounter earlier scientific publications on the effect of tDCS on cognitive functions that were in contradiction to the LU condition, when only sham tDCS would have been applied in that condition. Third, as the actively operating tNILS can induce qualitatively and quantitatively different cutaneous sensations, we decided to use only the sham tDCS protocol in both conditions as an active sham stimulation, in order to keep stimulation- generated cutaneous sensation constant between the sessions. Thus, the different treatment characteristics for the HU and LU conditions (tDCS + tNILS vs. tDCS only), were necessary due to the triple-blind nature of the study design, as operator blindness could not have been maintained under a full randomization of the stimulation devices.

### Paradigm

A probabilistic learning and decision-making task was used, which is a well-characterized task in humans^39^,^41^,^42^. It consists of a learning phase and a subsequent decision-making phase (see Fig. 1). During the learning phase, participants are repeatedly presented with three pairs (AB, CD and EF) of Chinese characters^42^. The participants’ task was to learn to choose the better option from a pair by trial and error. After each decision, the participants received feedback, and unknown to the participants, each symbol was probabilistically associated with reward (A – 80%, B – 20%; C – 70% D – 30%; E – 60% and F – 40%). The learning phase consisted of six experimental blocks; each block containing 20 repetitions of AB, CD and EF pairs in a random order. The presentation of each symbol pair was counterbalanced for the left and right side for each pair in each block (i.e., AB or BA).

Each trial started with a fixation cross (0.3), which was followed by the presentation of the symbol pair. Participants had a maximum of 1.7 s to respond, after which their choice was highlighted (0.5 s), and followed by the feedback (0.5 s). Each trial lasted for 3.5 s. In order to keep the motivational level constant between the treatment conditions, the participants were informed that they would receive 5 EUR per every started hour, plus 0.01 EUR after each correct decision (indicated by a green smiley face and a 0.01 EUR sign) or 0 EUR after an incorrect response (red, sad face and 0 EUR sign) or after a missed response (yellow confused face and the word ‘late’ in German). The test phase consisted of decisions involving all 15 possible combinations of the symbols; 12 new ones that were not presented during learning (e.g., AC or BF) as well as the three “old” combinations (AB, CD and EF). The symbol pairs were repeated 12 times resulting in a total of 180 decisions, and each symbol pair was counterbalanced for left and right side presentation. No feedback was provided during the testing phase, but the participants were informed that they would receive 0.01 EUR after each correct decision.. Taken together, in all three conditions (baseline, LU and HU), participants were motivated equally by performance-dependent financial remuneration. This procedure ensured that participants’ goal was to maximize their financial earnings.

After completing the final experimental session, the participants were informed about the amount of the financial compensation for their time and their additional earnings during the experiment. However, unknown to both participants and the operator, the final amount of the reimbursement was individually calculated according to 8.5 EUR per every started hour.

### Data analytic strategy

For data analysis, we only used Bayesian methods because of their many advantages compared to traditional null-hypothesis testing in general^48^,^49^ and in particular because they allow a hierarchical integration of the computational reinforcement-learning model^50^. We report our results in terms of posterior mean and the 95% highest-density intervals (HDIs), which give the range in which the estimated parameter is located with probability 0.95.

### Model-fitting

All reported models were fitted using Hamiltonian Monte-Carlo (HMC) techniques. We sampled from the joint posterior distribution of the parameters given the model using the HMC algorithms implemented in the Stan software^75^,^76^,^77^. All fits used eight parallel chains, each with a warm-up period of 1000 samples. Chains were initialized at random values and we sampled 1000 samples from each of the converged chains. We used no thinning as this was not deemed necessary by visual inspection of the chains and autocorrelation statistics. Resulting samples for each individual variable were visually inspected for convergence to ensure good mixing behaviour. We also applied the Gelman-Rubin diagnostic^78^ and ensured that all reported results had 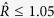. We used the Watanabe information criterion (WAIC) which resolves several of the difficulties of the deviance information criterion^79^,^80^,^81^. Differences in WAIC larger than 10 can be considered strong^82^.

### Analysis of performance data

We analysed learning performance by submitting the accuracy data from the learning part of the experiment to a Bayesian hierarchical logistic regression model. We included trial number, condition (baseline; low-expectancy, LU; high-expectancy, HU; dummy-coded) and pair number (1, 2, 3; coded numerically) as predictors and let the intercept vary by subject. Specifically, the accuracy in trial *t* for subject *i* was modeled as


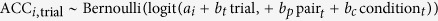


where the intercepts were constrained by a group-level distribution


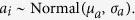


Non-informative (uniform) priors were placed on all variables. In words, the probability to respond correctly in a given trial was modeled as a function of the number of the current trial (because we expect performance to increase over the experiment as the subject learns the correct mapping), the pair number (the pairs had different reward probabilities and pairs with lower reward probabilities should therefore be harder to learn), the condition (reflecting potential placebo effects of the inert intervention) and the intercept accounting for between-subject variation in learning efficiency.

A similar analysis, using the same set of predictors, was applied to the reaction times (RTs). Specifically, the model was


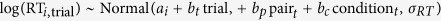


where the intercepts were constrained by a group-level distribution


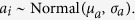


Non-informative (uniform) priors were placed on all variables. We used log-transformed reaction times because reaction times commonly have a non-normal distribution with a steep front and long tails, and are more appropriately modeled by a log-normal distribution^83^ but the results are qualitatively similar for non-transformed RTs.

### Analysis of subjective measures

In both experimental conditions, the participants were asked to anticipate whether the (inert) stimulation would improve, impair or not affect their performance relative to the baseline task. Similarly, they were asked immediately after the task whether they felt that their performance had been impaired, improved or not affected. We subjected the resulting categorical data for the anticipated and experienced effect of the stimulation for subject *i* to a softmax-regression model^49^:


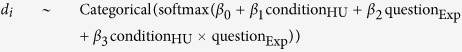


where condition_HU_ is an indicator variable which is 0 in the LU condition and 1 in the HU condition, question_Exp_ is the indicator variable for the two different questions (anticipated vs. experienced change in performance; 0 = Anticipated, 1 = Experienced) and *β* is a 4 × 3 matrix of parameters (intercept, condition, question, condition × question vs. decline, neutral, improve). We set “neutral” as reference category and fix 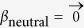 to make the parameter set identifiable. The softmax-function for a vector *θ* is defined as


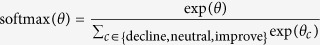


and yields a vector *p* = (*p*_decline_, *p*_neutral_, *p*_improve_) whose components are constrained to sum to one and represent the probabilites for each category.

To exclude the possibility that our results reflect the effect of a general arousal due to the presence of an intervention rather than a genuine placebo effect, we asked our participants to rate their arousal before and after each experimental session. The rating was done on an 11-point Likert scale where 0 indicated very tired and 10 fully awake. We submitted the resulting data to a Bayesian hierarchical linear regression model with condition (baseline, LU, HU) and time (before vs. after) and their interaction as variables:


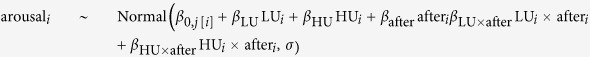


where the intercepts were constrained by a group-level distribution


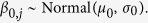


Here, “LU”, “HU”, and “after” are indicator variables that are 0 or 1 depending on the condition and time. In addition, *j*[*i*] indicates from which subject datapoint *i* was recorded. Non-informative (uniform) priors were placed on all parameters.

### Reinforcement-learning model

We used a fully Bayesian hierarchical modeling approach for fitting the QLearning model to the individual data. This approach has been successfully used previously in fitting reinforcement-learning models^50^ and is superior to classical, maximum-likelihood based inference for a number of reasons see, e.g., ref. 49: Firstly, it includes the likelihood of the data of all subjects in all conditions under a single comprehensive model. This allows for a very flexible choice of distributional assumptions and dependencies of the parameters. Second, the hierarchical structure enables a “partial pooling” of the individual estimates, resulting in more stable estimates^48^.

We implemented a standard QLearning model^39^,^84^. The accuracy data for each trial 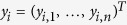 of subject *i* was modeled as as


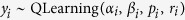


where the *n*-vector *p*_*i*_ ∈ {1, 2, 3}^*n*^ indicates the number of the pair that was shown to subject *i* in each trial and *r*_*i*_ ∈ {0, 1}^*n*^ is the vector indicating whether or not the subject received a reward in each trial (0-no reward, 1-reward present). The two parameters *α* and *β* are the learning rate and softmax-noise parameter, respectively. The learning-rate *α* influences the likelihood of the model by accumulating the expected value of all stimuli *s* ∈ {*A, B, C, D, E, F*} in the so-called *Q*-values


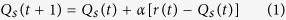


where *t* indexes trials 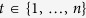. Here, *r(t*) − *Q*_*s*_(*t*) is the so-called prediction error and is slowly integrated into the general estimated reward-probability depending on the size of the learning-rate *α* ∈ [0, 1]. To account for uncertainty in the choice-reward mapping, the actual choice in the model is done probabilistically as well, where option *A* is chosen over option *B* in trial *t* with probability


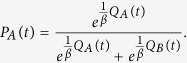


This is the so-called soft-max rule where the uncertainty in making a choice (or “noise”) is captured in the inverse-temperature parameter *β*. The likelihood is calculated as the product of these probabilities for each trial.

For each participant *i*, separate parameters *α*_*i*_ and *β*_*i*_ are estimated and they are constrained by group-level distributions


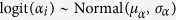


and


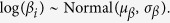


We were mainly interested in how our placebo manipulation would affect the model parameters and we therefore modeled the influence of the manipulation by adding them as fixed effects to the individual parameters:


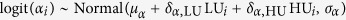


where LU_*i*_ and HU_*i*_ are indicator variables specifying whether the current dataset *i* (iterating over the subject × condition space) was acquired in condition LU or HU, respectively. Similarly,


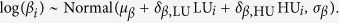


For a fully Bayesian approach, we had to specify prior distributions to the group-level parameters {*μ*_*α*_, *μ*_*β*_, *σ*_*α*_, *σ*_*β*_, *δ*_*α*,LU_, *δ*_*α*,HU_, *δ*_*β*,LU_, *δ*_*β*,HU_}. We assigned mildly informative priors to the parameters such that the parameter estimates were allowed to vary across a large number of parameter values while constraining them to a plausible range^48^,^85^. The results were robust to the choice of prior. During the model fitting, we experimented with different “degrees of non-informativeness” by changing the standard-deviation and limits of the prior distributions but the results were unchanged. Concretely, the reported analyses used the following prior


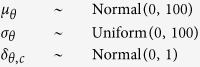


where *θ* ∈ {*α, β*} and *c* ∈ {LU, HU}.

As discussed above, we implemented a model where separate learning rates for learning from gains and learning from losses were estimated^39^ as learning from positive and negative outcomes appears to be implemented in by different sectors of striatal neurons. Concretely, equation (1) becomes


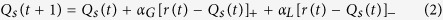


where [*x*]_+_ is *x* if positive and 0 otherwise and [*x*]_−_ is *x* if negative and 0 otherwise. All other elements of the hierarchical model (including the prior) remained identical, except that all group-level distributions and effects were calculated for *α*_*G*_, *α*_*L*_ and *β* independently.

## Additional Information

**How to cite this article**: Turi, Z. *et al*. Placebo Intervention Enhances Reward Learning in Healthy Individuals. *Sci. Rep.* **7**, 41028; doi: 10.1038/srep41028 (2017).

**Publisher's note:** Springer Nature remains neutral with regard to jurisdictional claims in published maps and institutional affiliations.

## Figures and Tables

**Table 1:** Estimated regression coefficients for performance data.

variable^a^	Accuracy	Reaction time^b^
trial^c^	0.144 [0.127, 0.160]^d^	−0.043 [−0.045, −0.041]
pair	−0.497 [−0.532, −0.463]	0.023 [0.019, 0.027]
condition LU	0.217 [0.146, 0.279]	−0.116 [−0.124, −0.107]
condition HU	0.435 [0.370, 0.503]	−0.145 [−0.153, −0.136]
intercept (group)	1.673 [1.389, 1.952]	−0.077 [−0.139, −0.014]

Placebo interventions resulted in more rapid performance with a smaller likelihood of errors.

^a^LU = low-uncertainty; HU = high-uncertainty.

^b^The model was fit to log-transformed reaction times.

^c^The effect of trial was rescaled to steps of 20 trials.

^d^Numbers indicate posterior mean and 95% HDI.

**Table 2:** Summary of the effects of the placebo intervention on the reinforcement-learning model parameters.

Variable	Condition	Mean, 95% HDI
effect on *α*_*G*_	LU	0.83 [0.55, 1.10]
	HU	1.17 [0.96, 1.39]
effect on *α*_*L*_	LU	−0.74 [−1.11, −0.36]
	HU	−0.86 [−1.37, −0.37]
effect on *β*	LU	−0.02 [−0.11, 0.07]
	HU	0.05 [−0.04, 0.14]

Placebo intervention increases dopamine-dependent learning from gains.

^a^*α*_*G*_ = learning rate from gains; *α*_*L*_ = learning rate from losses; *β* = softmax noise-parameter.

^b^LU = low-uncertainty; HU = high-uncertainty.

**Table 3:** Results of the Bayesian softmax-regression model for subjective expectation/experience.

Parameter	Expected/Experienced effect	Posterior distribution		
		Mean^a^	sd^b^	95% HDI^c^
intercept	negative	−1.72	0.87	[−3.49, −0.11]
	positive	0.60	0.43	[−0.23, 1.45]
condition	negative	0.50	1.13	[−1.66, 2.77]
	positive	−0.06	0.61	[−1.25, 1.15]
question	negative	−1.53	1.57	[−4.72, 1.50]
	positive	−0.92	0.59	[−2.10, 0.21]
condition × question	negative	2.02	1.79	[−1.41, 5.64]
	positive	−0.51	0.89	[−2.25, 1.24]

Coefficients denote changes in log-odds relative to “neutral”. There is no apparent effect of the placebo intervention on anticipated or experienced performance.

^a^Mean of the posterior distribution marginalized for each variable.

^b^Standard deviation of the marginal posterior distribution

^c^HDI = highest density interval.
